# Effect of GLP-1 Receptor Agonists in Heart Failure with Preserved Ejection Fraction: A Systematic Review and Meta-Analysis

**DOI:** 10.3390/jcdd13020103

**Published:** 2026-02-21

**Authors:** Benjamin J. Behers, Christian Sanchez, Omar Hozayen, Yousef Hozayen, Rheiner Kammer, William T. Corrigan, Christoph A. Stephenson-Moe, Matthew W. Miller, Mohab Idriss, Luis E. Cekan, Alan D. King, Garrett H. Brown, Karen M. Hamad

**Affiliations:** 1 Florida State University Internal Medicine Residency at Sarasota Memorial Hospital, Sarasota, FL 34239, USA; omar-hozayen@smh.com (O.H.); yousef-hozayen@smh.com (Y.H.); rheiner-kammer@smh.com (R.K.); william-corrigan@smh.com (W.T.C.); cstephensonmoe@med.fsu.edu (C.A.S.-M.); matthew-miller@smh.com (M.W.M.); mohab-idriss@smh.com (M.I.); alan-king@smh.com (A.D.K.); karen-hamad@smh.com (K.M.H.); 2 Department of Clinical Sciences, College of Osteopathic Medicine, Nova Southeastern University, Davie, FL 33328, USA; cs3653@mynsu.nova.edu; 3 Department of Clinical Sciences, College of Medicine, Florida State University, Tallahassee, FL 32304, USA; lec20e@med.fsu.edu; 4 Heart Specialists of Sarasota, Sarasota, FL 34239, USA; garretthbrown5@gmail.com

**Keywords:** GLP-1 RA, HFpEF, heart failure

## Abstract

Heart failure with preserved ejection fraction (HFpEF) affects 32 million people worldwide and is responsible for tens of billions of dollars in healthcare expenditure annually, with costs primarily driven by hospitalizations. HFpEF is notoriously difficult to treat, but emerging studies suggest that glucagon-like peptide-1 receptor agonists (GLP-1 RAs) may be effective therapies. We performed a systematic review and meta-analysis of six randomized controlled trials with 5564 total participants investigating GLP-1 RAs in patients with HFpEF. Overall, no significant effect was noted for GLP-1 RAs on our primary outcomes of cardiovascular mortality and worsening heart failure (HF) events, although they were associated with improvement in quality of life measures. Furthermore, safety data favored the GLP-1 RA group, although tolerability did not differ compared with placebo. While the pooled analysis of all GLP-1 RAs showed neutral effects versus hard endpoints, sensitivity analyses excluding older-generation agents (exenatide) revealed a significant 41% reduction in HF events, suggesting that newer, more potent agents (semaglutide, tirzepatide) may offer disease-modifying benefits in HFpEF. Although future studies are needed, GLP-1 RAs appear to be promising for the treatment of HFpEF.

## 1. Introduction

Heart failure with preserved ejection fraction (HFpEF) affects an estimated 32 million people worldwide and is responsible for tens of billions of dollars in healthcare expenditure annually, with costs primarily driven by hospitalizations [1,2]. Patients with HFpEF are hospitalized an estimated 1.4 times per year, with an annual mortality rate of approximately 15% and five-year mortality rates after hospitalization ranging from 50% to 75% [1]. Studies show that HFpEF now accounts for up to 50% of all hospitalizations for heart failure (HF), a number that is expected to continue increasing due to an aging global population and increased prevalence of coexisting conditions [2,3]. These coexisting conditions include obesity, type 2 diabetes mellitus, hypertension, atrial fibrillation, and chronic kidney disease [3]. HFpEF is characterized by a left ventricular ejection fraction (LVEF) of ≥50% with evidence of left ventricular diastolic dysfunction or elevated left ventricular filling pressures on echocardiogram [2,3]. It is caused by multiple complex pathophysiological mechanisms including myocardial fibrosis, hypertrophy, microvascular rarefaction, cardiac aging, and cardiometabolic disorders [3,4]. 

Contributing to its significant morbidity and mortality, as well as its global healthcare burden, is the fact that HFpEF is notoriously difficult to treat. Initially, management focused on diuretics to improve symptoms alongside treatment of the underlying cause, such as antihypertensives for HFpEF from hypertension-induced left ventricular hypertrophy. These approaches were used in spite of the fact that randomized controlled trials (RCTs) were unable to show any reduction in mortality or hospitalizations [3]. However, recent studies have yielded significant results, leading to recommendations for their use in patients with HFpEF. Sodium-glucose cotransporter 2 inhibitors (SGLT2is) have shown efficacy, with empagliflozin and dapagliflozin reducing the composite outcome of death from cardiovascular causes and hospitalization for heart failure in the 2021 EMPEROR-Preserved and 2022 DELIVER Trials, respectively [5,6]. Due to these findings, SGLT2is are now recognized as first-line treatment for HFpEF.

Emerging new trials have shown promising results from glucagon-like peptide-1 receptor agonists (GLP-1 RAs) in HFpEF [7]. However, this remains an ongoing area of research, and no formal treatment recommendations have come from these results. Given these findings, coupled with the significant societal impact of HFpEF, further elucidation of this effect is important. This study seeks to perform a meta-analysis of published RCTs reporting on the effect of GLP-1 RAs in patients with HFpEF, specifically investigating their effect on cardiovascular mortality (CV mortality) and worsening heart failure events (HF events).

## 2. Methods

This study was prospectively registered with PROSPERO (ID: CRD420251010277). It is presented in accordance with the Preferred Reporting Items for Systematic reviews and Meta-Analyses (PRISMA) guideline [8].

### 2.1. Eligibility Criteria

Studies were included if they (1) were placebo-controlled RCTs, (2) reported on the effect of GLP-1 RAs in patients with HFpEF, and (3) included outcome data on at least CV mortality and HF events. 

Studies were excluded if they did not (1) follow an RCT design, such as observational studies, (2) focus on population of patients with HFpEF or provide data for this group separately for extraction, and/or (3) include outcome data surrounding CV mortality and HF events.

### 2.2. Information Sources and Search Strategy

Embase, MEDLINE (PubMed), Web of Science, and Cochrane were systematically searched on 20 March 2025 for placebo-controlled RCTs examining the effects of GLP-1 RAs on patients with HFpEF (Table 1). The reference lists of the included studies were also reviewed to search for additional eligible studies.

### 2.3. Selection Process

Results of the systematic search were uploaded to Covidence (Veritas Health Innovation, Melbourne, Australia), a systematic review management software [9]. Two authors independently screened all studies for their adherence to eligibility criteria, initially by their title/abstract and then using the full text. A third author was available to solve disagreements during the screening process that could not be solved through discussion.

### 2.4. Data Collection Process and Data Items

Two authors independently extracted the basic characteristics of the included studies and data surrounding the primary and secondary endpoints. The basic characteristics included name of the trial, year of publication, sample size of both treatment and placebo groups, baseline patient demographics, baseline functional status, HFpEF definition for inclusion in the trial, GLP-1 RA used with dosage, and follow-up time of the trial. Baseline patient demographics consisted of age, percentage of female participants, body mass index (BMI), past medical history, and medications. Baseline functional status included New York Heart Association (NYHA) Class, Kansas City Cardiomyopathy Questionnaire Clinical Summary Score (KCCQ-CSS), and 6 min walk test (6-MWT) distance. Data surrounding the primary and secondary endpoints included sample sizes for both treatment and placebo groups, number of events in each group for categorical outcomes, and mean with standard deviation for continuous outcomes.

### 2.5. Study Risk of Bias Assessment

Quality assessment of included studies was performed using the Risk of Bias 2 tool [10]. Risk of bias was examined based on five domains: (1) the randomization process, (2) deviations from planned interventions and study intent, (3) missing or omitted outcome data, (4) the method for measurement of outcomes, and (5) selection of the reported outcome. Each domain was scored either (1) low risk, (2) some concerns, or (3) high risk. Two authors independently performed this assessment for each included study, with a third author available to solve any disagreements.

### 2.6. Effect Measures and Synthesis Methods

Two primary outcomes were investigated separately: CV mortality and worsening HF events. Worsening HF events were defined as hospitalization or an urgent care visit for HF. We thoroughly analyzed the included studies to ensure our primary outcomes were carefully adjudicated, including reviewing Supplementary Files. Secondary outcomes included all-cause mortality, a change in KCCQ-CSS score from baseline, a change in 6-MWT score from baseline, serious adverse events (SAEs), and serious adverse events requiring medication discontinuation (SAE-MDs). CV mortality, HF events, all-cause mortality, SAE, and SAE-MD were all reported as categorical variables, while KCCQ-CSS and 6-MWT were reported as continuous variables. 

Overall effect sizes were calculated using the random effects model and inverse variance method, with its 95% confidence interval (95% CI) calculated using the Wald-type method [11]. Risk ratio (RR) was used for categorical variables, while mean difference (MD) was used for continuous variables. Heterogeneity was assessed using Tau^2^, the Chi^2^ test, and the *I*^2^ statistic. Tau^2^ calculated the between-study variance using the restricted maximum likelihood method [12]. A statistical analysis for heterogeneity was performed using the Chi^2^ test at a significance level of 0.1 [13]. Percentage of variation across studies due to variance was assessed using the *I*^2^ statistic, with 0–30% representing low heterogeneity, 30–60% moderate, and >60% high [14]. Sensitivity analyses were performed using the leave-one-study-out method [15]. Statistical analysis was performed using RevMan Version 9.17.0 [16].

### 2.7. Reporting Bias Assessment

Reporting bias was assessed across the primary outcomes by using funnel plots and assessing for asymmetry [17]. Two authors independently assessed these funnel plots, with a third author available to solve disagreements.

## 3. Results

### 3.1. Study Selection

**Figure 1:**
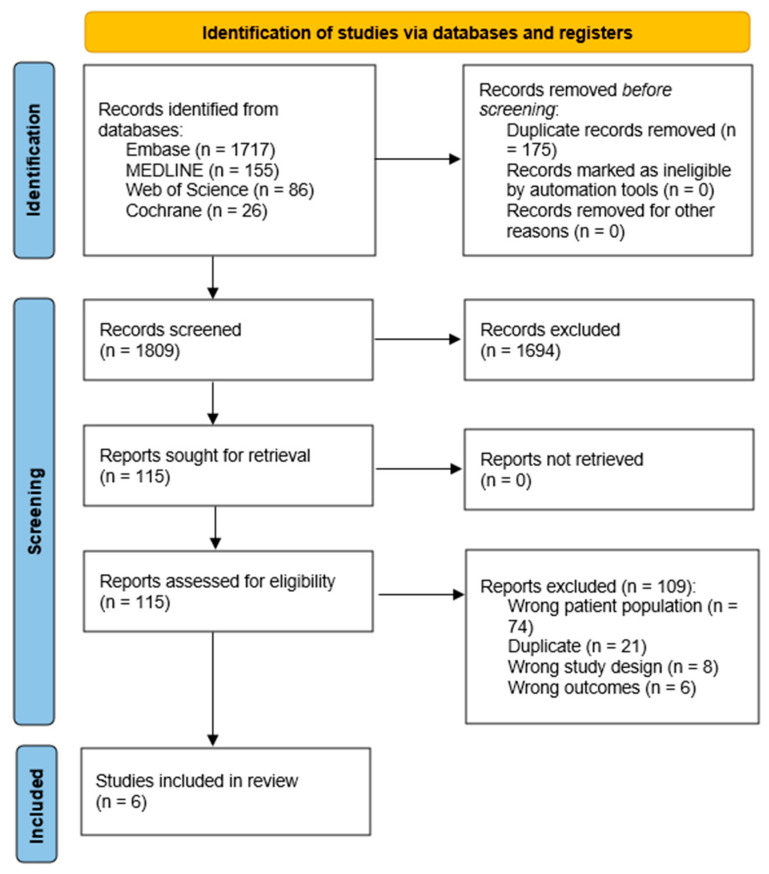
PRISMA flowchart detailing the study selection process.

There were 1984 studies identified across the four databases: Embase (*n* = 1717), MEDLINE (*n* = 155), Web of Science (*n* = 86), and Cochrane (*n* = 26). Covidence automatically removed 175 duplicates, leaving 1809 studies for title/abstract screening. Title/abstract screening resulted in removal of 1694 studies, followed by assessment of the full texts of the remaining 115 studies for eligibility. Ultimately, six studies met the eligibility criteria and were included in this study (Figure 1).

### 3.2. Study Characteristics

Six studies met the inclusion criteria and were published from 2019 to 2025 [18,19,20,21,22,23]. There were 5564 total participants, with median ages ranging from 61.7 to 70.0 years, with most trials exhibiting male predominance. The median body mass index (BMI) of participants ranged from 32.8 to 38.3 kg/m^2^, and they had various co-morbidities, with hypertension and type 2 diabetes mellitus being the most common, ranging from 0 to 100% and 81.6 to 99.1%, respectively. Participants were also on numerous other medications, with angiotensin-converting enzyme inhibitor or angiotensin receptor blocker (ACE/ARB) and beta-blockers being the most common, ranging from 79.8 to 96.0% and 67.3 to 82.9%, respectively. Use of SGLT2is was reported in five trials and ranged from 0 to 34.5%. Across all trials, the majority of participants had NYHA class II symptoms, while baseline KCCQ-CSS and 6-MWT distance were only reported in three trials. HFpEF was defined as LVEF ≥ 40% in three studies, ≥45% in two studies, and ≥50% in one. The GLP-1 RAs utilized in the included studies were semaglutide in four trials, exenatide in one trial, and tirzepatide in one trial. Follow-up times for the trials ranged from 39.8 months to 3.4 years (Table 2).

### 3.3. Risk of Bias in Studies

**Figure 2:**
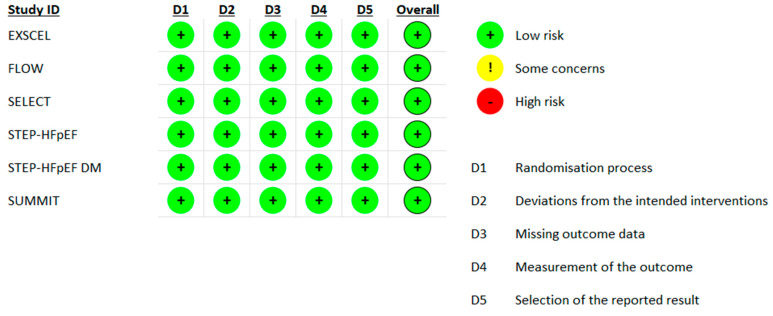
Risk of bias in included studies [18,19,20,21,22,23].

Using the Cochrane Risk of Bias 2.0 tool, all included trials were ultimately judged to be at low risk of bias across domains. In the EXSCEL and FLOW trials, HF events were investigator-reported without central adjudication; however, outcome definitions were prespecified, blinding was maintained, and these features were not judged to materially compromise outcome measurement at the trial level [18,19]. In SELECT and SUMMIT, protocol amendments or exploratory analyses of individual components of prespecified composite outcomes were noted, but these changes were finalized prior to unblinding, transparently reported, and conducted according to predefined statistical analysis plans [20,23]. Across all studies, randomization procedures were robust, deviations from intended interventions were minimal, outcome data were largely complete, and no evidence of selective reporting was identified, supporting an overall evaluation of low risk of bias (Figure 2).

### 3.4. Results of Individual Studies and Results of Syntheses

**Figure 3:**
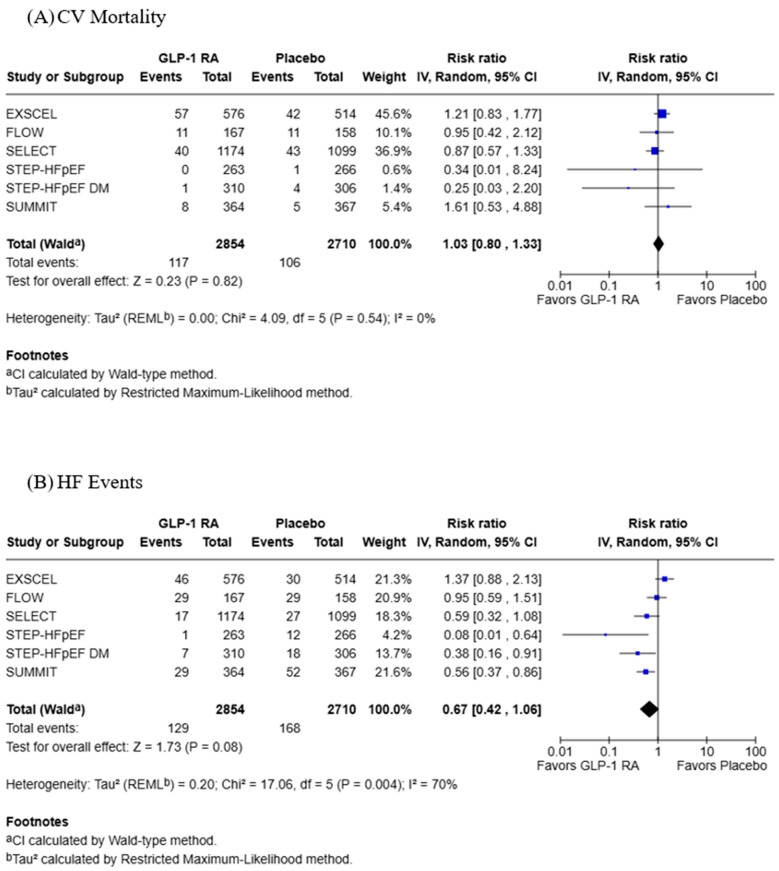
Forest plots for (**A**) CV mortality and (**B**) HF events [18,19,20,21,22,23].

All six studies included data for CV mortality and worsening HF events with 2854 participants in the GLP-1 RA group and 2710 in the placebo group (Figure 3). GLP-1 RAs had no significant effect on CV mortality (RR: 1.03; 95% CI: 0.80, 1.33; *p* = 0.82), and this analysis had low heterogeneity (Tau^2^ = 0.00; Chi^2^ = 4.09, df = 5, *p* = 0.54; *I*^2^ = 0%). GLP-1 RAs also had no significant effect on HF events (RR: 0.67; 95% CI: 0.42, 1.06; *p* = 0.08), although high heterogeneity was observed with this analysis (Tau^2^ = 0.20; Chi^2^ = 17.06, df = 5, *p* = 0.004; *I*^2^ = 70%).

**Figure 4:**
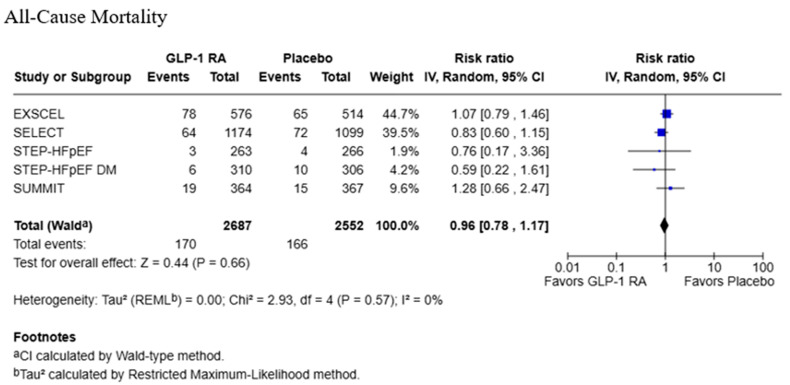
Forest plot for all-cause mortality [18,20,21,22,23].

All-cause mortality was reported in five studies with 2687 participants in the GLP-1 RA group and 2552 in the placebo group (Figure 4). There was no significant effect from GLP-1 RA on all-cause mortality (RR: 0.96; 95% CI: 0.78, 1.17; *p* = 0.66), with low heterogeneity (Tau^2^ = 0.00; Chi^2^ = 2.93, df = 4, *p* = 0.57; *I*^2^ = 0%). 

**Figure 5:**
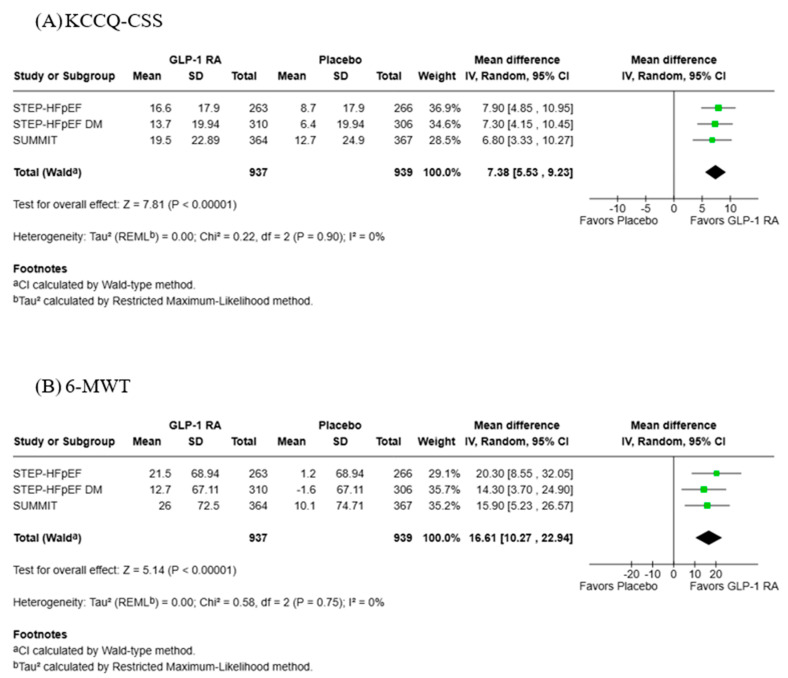
Forest plots for (**A**) Change in KCCQ-CSS and (**B**) 6-MWT distance [21,22,23].

Data for change in KCCQ-CSS and 6-MWT distance was reported in three studies with 937 participants in the GLP-1 RA group and 939 in the placebo group (Figure 5). GLP-1 RAs were associated with a statistically significant increase in KCCQ-CSS score (MD: 7.38; 95% CI: 5.53, 9.23; *p* = <0.00001) with low heterogeneity (Tau^2^ = 0.00; Chi^2^ = 0.22, df = 2, *p* = 0.75; *I*^2^ = 0%). GLP-1 RAs were also associated with a statistically significant increase in 6-MWT distance (MD: 16.61 m; 95% CI: 10.27, 22.94; *p* = <0.00001) with low heterogeneity (Tau^2^ = 0.00; Chi^2^ = 0.58, df = 2, *p* = 0.75; *I*^2^ = 0%). 

**Figure 6:**
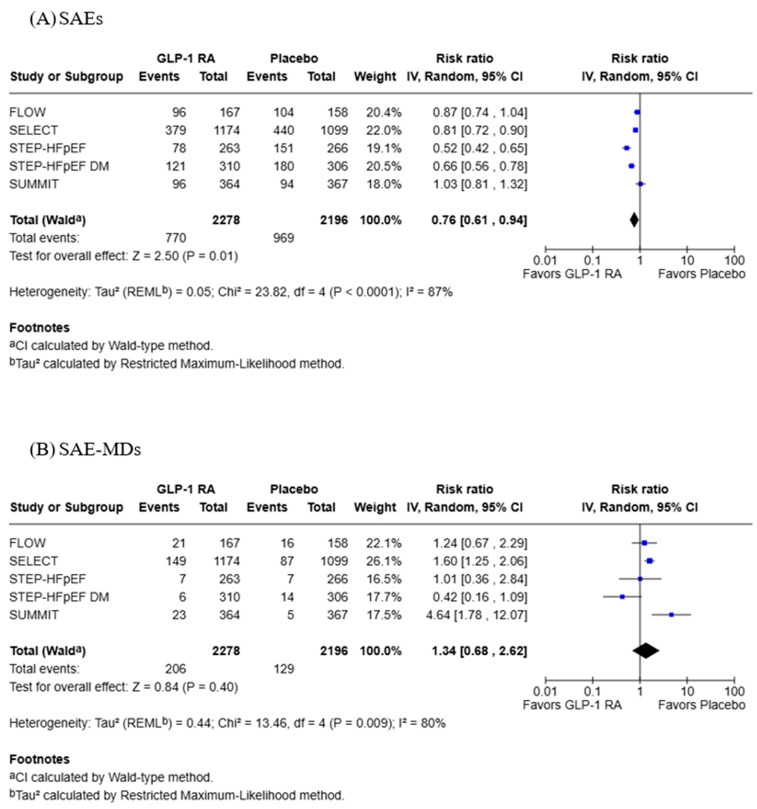
Forest plots for (**A**) SAEs and (**B**) SAE-MDs [19,20,21,22,23].

Data reporting SAEs and SAE-MDs was reported in five studies with 2278 participants in the GLP-1 RA group and 2196 in the placebo group (Figure 6). GLP-1 RAs were associated with statistically significant fewer SAEs (RR: 0.76; 95% CI: 0.61, 0.94; *p* = 0.01), although this analysis had high heterogeneity (Tau^2^ = 0.05; Chi^2^ = 23.82, df = 4, *p* = <0.0001; *I*^2^ = 87%). There was no significant difference between groups in regards to SAE-MDs (RR: 1.34; 95% CI: 0.68, 2.62; *p* = 0.40), and this analysis also had high heterogeneity (Tau^2^ = 0.44; Chi^2^ = 13.46, df = 4, *p* = 0.009; *I*^2^ = 80%). 

Sensitivity analyses can be seen in Table S1. Removal of the EXSCEL trial led to our HF event outcome becoming significant, with GLP-1 RAs associated with a statistically significant reduction in HF events (RR: 0.59; 95% CI: 0.41, 0.86; *p* = 0.005). Removal of the STEP-HFpEF DM trial led to our SAE outcome no longer being significant, with no difference between groups (RR: 0.78; 95% CI: 0.60, 1.03; *p* = 0.08). Removal of the STEP-HFpEF DM trial also led to our SAE-MD outcome becoming significant, with GLP-1 RAs being associated with a statistically significant increase in SAE-MDs (1.66; 95% CI: 1.07, 2.57; *p* = 0.02).

### 3.5. Reporting Biases

Funnel plots for our primary outcomes of CV mortality and HF events can be seen in Figure S1. No asymmetry is noted in the funnel plot for CV mortality, suggesting a low risk of publication bias. However, asymmetry is noted in the funnel plot for HF events, suggesting the possibility of publication bias.

## 4. Discussion

Our results indicate that, compared with placebo, GLP-1 RAs have no significant effect on CV mortality, worsening HF events, or all-cause mortality. Despite these neutral findings on hard endpoints, GLP-1 RAs were associated with meaningful improvements in quality of life, as reflected by higher KCCQ-CSS scores and 6-MWT distances. Safety outcomes favored the GLP-1 RA group, with fewer serious adverse events overall, whereas tolerability was comparable between groups, as evidenced by similar rates of discontinuation due to adverse events. Discontinuation in the GLP-1 RA group was driven primarily by gastrointestinal side effects, a known limiting factor in their use. However, heterogeneity in our HF events analysis was high at 70%, and four of our six included studies favored GLP-1 Ras, with three reaching independent significance, emphasizing importance of performing sensitivity analyses. Notably, removal of the EXSCEL trial in sensitivity analyses rendered the difference in HF events significant, showing a 41% reduction in events with GLP-1 RAs versus placebo. This result is consistent with our broader understanding of drug heterogeneity within this class. EXSCEL, which evaluated exenatide, was the only included trial demonstrating more HF events in the active treatment arm, contributing to the observed heterogeneity.

Given the observed heterogeneity, exploring differences between various GLP-1 RAs is important. Exenatide was the first GLP-1 RA approved back in 2005, while semaglutide and tirzepatide were approved in 2019 and 2022, respectively [24]. One study found that semaglutide reduced the risk of major adverse cardiovascular events (CV death, non-fatal stroke, and non-fatal myocardial infarction) against its comparators, which included exenatide, dulaglutide, and liraglutide [25]. A network meta-analysis of GLP-1 RAs in HFpEF ranked tirzepatide highest in reducing hospitalizations for heart failure, while semaglutide ranked highest in reducing a composite of CV mortality and worsening HF events, as well as improving the 6-MWT distance [26]. These newer GLP-1 RAs are thought to achieve better effects due to greater potency and longer half-lives due to structural modifications that resist degradation by DPP-4 [27]. Additionally, tirzepatide is a dual GLP-1 RA and glucose-dependent insulinotropic polypeptide (GIP) RA, with activation of both receptors resulting in superior metabolic and hemodynamic effects [27]. These structural modifications in newer GLP-1 RAs, such as semaglutide, and dual activation of tirzepatide likely explain why our HF event data was largely driven by the STEP-HFpEF, STEP-HFpEF DM, and SUMMIT trials.

These findings are further corroborated in a meta-analysis of three RCTs investigating semaglutide (STEP-HFpEF and STEP-HFpEF DM) and tirzepatide (SUMMIT) on obesity-related HFpEF, all three of which were included in our study [28]. They found a statistically significant 60% reduction in HF events but no significant difference in CV mortality [28]. Furthermore, in contrast to our study, they found increased rates of discontinuation due to adverse events in the GLP-1 RA group, primarily due to gastrointestinal side effects [28]. However, interestingly, another meta-analysis utilizing the same six RCTs as our study yielded conflicting results, with use of GLP-1 RAs resulting in a significant reduction in worsening HF events, although they also found no significant reduction in CV mortality [29]. Their use of the hazard ratio compared to our risk ratio suggests the time to HF event may explain this variance. Another meta-analysis of four studies consisting of 1463 patients found that semaglutide resulted in a significant increase in the 6-MWT distance of 16.20 m from baseline, similar to our value of 16.61 m [30].

Cohort studies have yielded similar findings to these meta-analyses. One study consisting of five cohort studies and 21,151 participants found that semaglutide and tirzepatide achieved a 40% risk reduction compared to placebo in the composite of hospitalization for HF and all-cause mortality [31]. Additionally, no significant differences were noted in event rates between semaglutide and tirzepatide [31]. Another study of 84,990 patients with HFpEF also found that GLP-1 RAs compared to placebo were associated with a 40% reduction in HF events and a 33% reduction in all-cause emergency room visits or hospitalizations [32]. A third study examining the effect of semaglutide on exercise capacity in 318 patients found a significant increase in the 6-MWT distance of 15.1 m compared to placebo [33]. Despite our insignificant results prior to sensitivity analyses, the amalgamation of data surrounding the potential of GLP-1 RAs to both reduce HF events and improve quality of life in HFpEF is difficult to ignore. Future studies should continue exploring this phenomenon with the newer GLP-1 RAs to further elucidate the true clinical effect.

It is also important to explore how GLP-1 RAs exert their observed cardiovascular benefits. GLP-1 RAs mimic endogenous GLP-1, which is an incretin involved in modulating glucose-dependent insulin secretion, suppressing glucagon release, and delaying gastric emptying [34]. The cardiovascular benefits of GLP-1 RAs are thought to be primarily due to indirect mechanisms, such as weight loss, blood pressure reduction, improved glycemic control, and anti-inflammatory effects [34]. However, some direct mechanisms have also been proposed, such as improved myocardial perfusion through endothelial modulation, antifibrotic effects on myocardial tissue, reduced epicardial adipose tissue, modulation of the autonomic nervous system towards parasympathetic activity, and through their promotion of natriuresis and diuresis [34]. Additionally, echocardiographic studies have shown improvement in cardiac remodeling from GLP-1 RAs in HFpEF, including increased chamber volumes and improved left ventricular diastolic function [35]. These increased chamber volumes and improved diastolic function are the likely mechanisms by which GLP-1 RAs reduce hospitalizations in HFpEF. Future studies into the mechanism by which GLP-1 RAs exhibit their improvements in HFpEF is warranted.

As highlighted in our introduction, SGLT2is are currently the first-line therapy for HFpEF. Concomitant use of SGLT2is with GLP-1 RAs was reported in five of our included studies and ranged from 0 to 34.5%, primarily in the STEP-HFpEF DM and SUMMIT trials, both of which contributed strongly to our HF event data. Studies investigating this use have yielded encouraging findings. For instance, a retrospective cohort study with 7044 participants in each group found that GLP-1 RAs combined with SGLT2is were associated with a significantly lower risk of heart failure hospitalizations than SGLT2is alone in patients with type 2 diabetes and obesity-related HFpEF [36]. Another study comparing GLP-1 RAs to SGLT2is in patients with type 2 diabetes and HFpEF found that GLP-1 RAs significantly reduced the risk of cardiovascular events, progression to systolic heart failure, and stroke [37]. Future studies should continue to explore these findings, and treatment recommendations for HFpEF should be updated accordingly.

Despite these promising findings, several limitations warrant consideration. First, the relatively small number of eligible randomized controlled trials restricts the precision of our estimates. Second, we pooled various GLP-1 RAs despite potential pharmacologic differences among agents; future analyses isolating individual drugs may yield more definitive insights. Furthermore, we did not control for the use of SGLT2is, which have also been shown to improve clinical outcomes in HFpEF. Third, meta-analyses are inherently susceptible to publication and selection bias, and variations in inclusion criteria, particularly left ventricular ejection fraction thresholds, may limit generalizability. Additionally, the accuracy of our analyses is reliant upon the reporting in our included studies, which also poses a risk of bias, especially for our HF event data. Lastly, subgroup analyses exploring heterogeneity by diabetes status, obesity, or sex were not performed but should be prioritized in future work.

## 5. Conclusions

Overall, our results show that GLP-1 RAs improve quality of life in patients with HFpEF but have no effect on hospitalizations or mortality. However, the findings of our sensitivity analysis and results from other studies suggest that newer GLP-1 RAs, such as semaglutide and tirzepatide, decrease heart failure hospitalizations. Although future studies are needed, GLP-1 RAs appear to be an effective therapy for HFpEF. 

## Figures and Tables

**Table 1:** Search strategies and number of results across the four databases.

Database	Results	Search Strategy
Embase	1717	1. GLP-1 RAs: (exp glucagon like peptide 1 receptor agonist/OR (“glp-1 receptor agonist” OR “glp-1 agonist” OR “incretin mimetic” OR “incretin-based therap” OR exenatide OR liraglutide OR dulaglutide OR semaglutide OR albiglutide OR lixisenatide OR Byetta OR Bydureon OR Victoza OR Trulicity OR Ozempic OR Wegovy OR Adlyxin OR Tanzeum OR Rybelsus).ti,ab,kw) 2. HFpEF: (exp heart failure, diastolic/OR “heart failure with preserved ejection fraction”.ti,ab,kw OR HFpEF.ti,ab,kw OR “heart failure with normal ejection fraction”.ti,ab,kw OR HFNEF.ti,ab,kw OR “diastolic heart failure”.ti,ab,kw OR “diastolic dysfunction”.ti,ab,kw) 3. Cardiovascular Outcomes: (exp cardiovascular disease/OR “cardiovascular outcome”.ti,ab,kw OR “CV outcome”.ti,ab,kw OR “cardiovascular event”.ti,ab,kw OR “CV event”.ti,ab,kw OR MACE.ti,ab,kw OR “major adverse cardiac event”.ti,ab,kw OR “cardiac mortality”.ti,ab,kw OR “cardiovascular mortality”.ti,ab,kw OR “heart failure hospitalization”.ti,ab,kw OR hospitalization.ti,ab,kw OR “hospital admission”.ti,ab,kw OR “hospital readmission”.ti,ab,kw OR mortality.ti,ab,kw OR death.ti,ab,kw) 4. RCT Filter: (exp randomized controlled trial/OR “randomized controlled trial”.ti,ab,kw OR “randomised controlled trial”.ti,ab,kw OR RCT.ti,ab,kw OR “controlled clinical trial”.ti,ab,kw OR “clinical trial”.ti,ab,kw OR “double-blind”.ti,ab,kw OR “single-blind”.ti,ab,kw OR placebo.ti,ab,kw OR “phase 2”.ti,ab,kw OR “phase II”.ti,ab,kw OR “phase 3”.ti,ab,kw OR “phase III”.ti,ab,kw OR trial.ti,ab,kw) Final Combination: 1 AND 2 AND 3 AND 4
MEDLINE (PubMed)	155	1. GLP-1 RAs: (“Glucagon-Like Peptide 1 Receptor Agonists”[Mesh] OR “GLP-1 receptor agonist” OR “GLP-1 agonist” OR “incretin mimetic” OR “incretin-based therap” OR exenatide OR liraglutide OR dulaglutide OR semaglutide OR albiglutide OR lixisenatide OR Byetta OR Bydureon OR Victoza OR Trulicity OR Ozempic OR Wegovy OR Adlyxin OR Tanzeum OR Rybelsus) 2. HFpEF: (“Heart Failure, Diastolic”[Mesh] OR “heart failure with preserved ejection fraction” OR HFpEF OR “heart failure with normal ejection fraction” OR HFNEF OR “diastolic heart failure” OR “diastolic dysfunction”) 3. Cardiovascular Outcomes: (“Cardiovascular Diseases”[Mesh] OR “cardiovascular outcome” OR “CV outcome” OR “cardiovascular event” OR “CV event” OR MACE OR “major adverse cardiac event” OR “cardiac mortality” OR “cardiovascular mortality” OR “heart failure hospitalization” OR hospitalization OR “hospital admission” OR “hospital readmission” OR mortality OR death) 4. RCT Filter: (“Randomized Controlled Trial”[Publication Type] OR “randomized controlled trial” OR “randomised controlled trial” OR RCT OR “controlled clinical trial” OR “clinical trial” OR “double-blind” OR “single-blind” OR placebo OR trial) Final Combination: 1 AND 2 AND 3 AND 4
Web of Science	86	1. GLP-1 RAs: TS = (“GLP-1 receptor agonist” OR “GLP-1 agonist” OR “incretin mimetic” OR “incretin-based therap” OR exenatide OR liraglutide OR dulaglutide OR semaglutide OR albiglutide OR lixisenatide OR Byetta OR Bydureon OR Victoza OR Trulicity OR Ozempic OR Wegovy OR Adlyxin OR Tanzeum OR Rybelsus) 2. HFpEF: TS = (“heart failure with preserved ejection fraction” OR HFpEF OR “heart failure with normal ejection fraction” OR HFNEF OR “diastolic heart failure” OR “diastolic dysfunction”) 3. Cardiovascular Outcomes: TS = (“cardiovascular outcome” OR “CV outcome” OR “cardiovascular event” OR “CV event” OR MACE OR “major adverse cardiac event” OR “cardiac mortality” OR “cardiovascular mortality” OR “heart failure hospitalization” OR hospitalization OR “hospital admission” OR “hospital readmission” OR mortality OR death) 4. RCT Filter: TS = (“randomized controlled trial” OR “randomised controlled trial” OR RCT OR “controlled clinical trial” OR “clinical trial” OR “double-blind” OR “single-blind” OR placebo OR trial) Final Combination: 1 AND 2 AND 3 AND 4
Cochrane	26	1. GLP-1 RAs: (“GLP-1 receptor agonist” OR “GLP-1 agonist” OR exenatide OR liraglutide OR dulaglutide OR semaglutide OR albiglutide OR lixisenatide OR Byetta OR Bydureon OR Victoza OR Trulicity OR Ozempic OR Wegovy OR Adlyxin OR Tanzeum OR Rybelsus) 2. HFpEF: (“heart failure with preserved ejection fraction” OR HFpEF OR “heart failure with normal ejection fraction” OR HFNEF OR “diastolic heart failure” OR “diastolic dysfunction”) 3. Cardiovascular Outcomes: (“cardiovascular outcome” OR “CV outcome” OR “cardiovascular event” OR “CV event” OR MACE OR “major adverse cardiac event” OR “cardiac mortality” OR “cardiovascular mortality” OR “heart failure hospitalization” OR hospitalization OR “hospital admission” OR “hospital readmission” OR mortality OR death) 4. RCT Filter: (“randomized controlled trial” OR “randomised controlled trial” OR RCT OR “controlled clinical trial” OR “clinical trial” OR “double-blind” OR “single-blind” OR placebo OR trial) Final Combination: 1 AND 2 AND 3 AND 4

**Table 2:** Basic characteristics of included studies.

Study	EXSCEL [18]		FLOW [19]		SELECT [20]		STEP-HFpEF [21]		STEP-HFpEF DM [22]		SUMMIT [23]	
Pub Yr	2019		2024		2024		2023		2024		2025	
N	T	P	T	P	T	P	T	P	T	P	T	P
	576	514	167	158	1174	1099	263	266	310	306	364	367
Age (Years)	64.0 (58.0–69.0)	63.5 (58.0–69.0)	69.0 (63.0–73.0)		61.7 (8.7) *		70 (62–75)	69 (62–75)	69.0 (62.0–74.0)	70.0 (63.0–75.0)	65.5 (10.5) *	65.0 (10.9) *
F (%)	35.0	36.1	45.8		31.1		56.7	55.6	41.3	47.4	54.9	52.6
BMI (kg/m^2^)	33.0 (29.3–37.5)	32.8 (29.4–37.3)	33.8 (30.1–37.9)		32.8 (30.1–36.7)		37.2 (33.9–41.1)	36.9 (33.3–41.6)	36.9 (33.6–41.5)	36.9 (33.5–41.1)	38.3 (6.4) *	38.2 (7.0) *
CAD (%)	72.4	72.7	71.4		NR		20.2	16.9	25.5	22.5	30.9	29.1
MI (%)	52.8	52.5	35.5		70.3		NR	NR	NR	NR	NR	NR
AF (%)	NR	NR	18.8		NR		51.3	52.6	37.7	41.2	26.1	48.5
HTN (%)	NR	NR	99.1		NR		82.1	81.6	82.3	88.6	NR	NR
DM2 (%)	100	100	100		0		0	0	100	100	47.8	48.5
ACE/ARB (%)	88.3	87.5	96.0		83.2		79.8	80.5	80.3	82.7	80.5	80.4
BB (%)	74.9	75.9	74.2		82.2		76.4	81.6	82.9	82.7	67.3	71.7
MRA (%)	19.0	17.1	14.8		17.8		33.8	35.7	33.9	31.0	36.0	34.1
SGLT2i (%)	NR	NR	11.4		0		3.0	4.1	34.5	31.0	19.0	15.5
NYHA I (%)	31.5	30.4	31.9		33.0		0	0	0	0	0	0
NYHA II (%)	54.1	57.5	57.4		59.0		69.6	62.8	71.9	69.3	72.0	73.0
NYHA III/IV (%)	14.4	12.1	9.8		8.0		30.4	37.2	28.1	30.7	28.0	27.0
KCCQ-CSS (Points)	NR	NR	NR		NR		59.4 (42.7–72.9)	58.3 (40.5–72.9)	60.4 (44.8–72.9)	58.3 (41.1–70.8)	53.9 (17.9) *	53.2 (19.0) *
6-MWT (Meters)	NR	NR	NR		NR		316.0 (251.0–386.0)	325.8 (232.4–392.0)	280.0 (205.1–357.6)	280.0 (200.0–345.0)	305.0 (80.0) *	300.6 (83.5) *
HFpEF Def. (%)	≥40		≥40		≥40		≥45		≥45		≥50	
GLP-1 RA	Exenatide 2 mg weekly		Semaglutide 1 mg weekly		Semaglutide 2.4 mg weekly		Semaglutide 2.4 mg weekly		Semaglutide 2.4 mg weekly		Tirzepatide 15 mg weekly	
F/U	Median 3.2 years		Median 3.4 years		Mean 39.8 months		52 weeks		52 weeks		52 weeks	

Pub Yr: signifies the year of publication for the study; N: sample size; T: treatment (GLP-1 RA) group; P: placebo group; F: female participants; BMI: body mass index (kg/m^2^); CAD: coronary artery disease; MI: prior myocardial infarction; AF: atrial fibrillation; HTN: hypertension; DM2: type 2 diabetes mellitus; ACE/ARB: angiotensin-converting enzyme inhibitor or angiotensin receptor blocker; BB: beta-blocker; MRA: mineralocorticoid receptor antagonist; SGLT2i: sodium-glucose cotransporter 2 inhibitors; NYHA I, II, and III/IV: percentage of participants with New York Heart Association functional classification class I, II, and III/IV symptoms; KCCQ-CSS: Baseline Kansas City Cardiomyopathy Questionnaire Clinical Summary Score (points); 6-MWT: baseline 6-min walk test distance (meters); HFpEF Def.: LVEF used to define HFpEF in the trial; GLP-1 RA: glucagon-like peptide-1 receptor agonist used in the trial; F/U: trial follow-up time. NR: Signifies that the data was not reported in the trial. Data is reported as median (IQR) and frequencies with percentages unless denoted otherwise. * Data is reported as mean (SD).

## Data Availability

Data is available from the first author upon reasonable request.

## Supplementary Materials

The following supporting information can be downloaded at: https://www.mdpi.com/article/10.3390/jcdd13020103/s1, Table S1: Results of sensitivity analyses using the leave-one-study-out method., Figure S1: Funnel plots for A) CV mortality and B) HF events., Table S2: PRISMA checklist.
